# Cascading 58mer Alpha Satellite superHOR in Complete Orangutan Y Chromosome

**DOI:** 10.3390/ijms26178122

**Published:** 2025-08-22

**Authors:** Matko Glunčić, Ines Vlahović, Marija Rosandić, Vladimir Paar

**Affiliations:** 1 Faculty of Science, University of Zagreb, 10000 Zagreb, Croatia; vpaar@hazu.hr; 2 Department of Interdisciplinary Sciences, Algebra University College, 10000 Zagreb, Croatia; ines.vlahovic@algebra.hr; 3 University Hospital Centre Zagreb, 10000 Zagreb, Croatia; rosandic@hazu.hr; 4 Croatian Academy of Sciences and Arts, 10000 Zagreb, Croatia

**Keywords:** orangutan centromere, human centromere, chimpanzee centromere, gorilla centromere, alpha satellite, higher order repeat HOR, GRMhor

## Abstract

Recent analyses have revealed that orangutan alpha satellite higher-order repeat (HOR) arrays in complete centromeres are composed of three to four distinct HOR blocks, each sharing only 80–90% sequence identity, thus forming a patchwork-quilt pattern of independent HOR expansions. In contrast, using our novel HOR-detection algorithm GRhor, we analyzed the complete Y chromosome centromere in orangutan and identified a highly ordered and complex alpha satellite 58mer superHOR array, comprising 67 HOR copies, including 46 highly identical canonical copies with a remarkably low divergence of only 0.25%. Given that the largest known human alpha satellite HOR is the 34mer on the Y chromosome, this novel 58mer structure qualifies as a superHOR. The canonical 58mer HOR contains only 44 distinct monomer types, with 14 types repeated within the unit, resulting in a unique five-row cascading organization. Such complexity is not detectable using standard HOR-searching tools employed in previous studies. Additionally, we identified a second, less pronounced 45mer cascading superHOR array with 0.81% divergence. For comparative purposes, we also detected a cascading 18mer HOR in gorilla and a Willard-type 28mer HOR in chimpanzee Y centromeres. Notably, preliminary genome-wide analysis in orangutan reveals other superHORs, including 84mer and 53mer arrays in chromosome 5; a 54mer in chromosome 10; a 51mer in chromosome 14; a 53mer in chromosome 15; and a 45mer in chromosome 22. These findings underscore the power of GRMhor in revealing highly structured and species-specific HOR architectures, with potential implications for centromere evolution and primate comparative genomics.

## 1. Introduction

The recent availability of complete centromere assemblies in humans and nonhuman primates has revealed an unexpected diversity in sequence composition, rapid evolutionary dynamics, and highly complex organizational patterns [1,2,3,4]. Until recently, only partial centromere sequences were available, which showed that centromeres are primarily composed of ~171 bp alpha satellite monomers organized into tandem arrays. These monomers are typically structured into HOR units consisting of *n* monomers, each of a distinct sequence type [5,6,7,8,9]. Such structured arrays are known as Willard-type HORs, in which each monomer within a HOR unit is unique [5,9,10].

Within each HOR copy, monomer sequences often exhibit substantial divergence, typically ranging from 20% to 40%. In contrast, HOR copies themselves tend to be more conserved, with sequence divergence usually less than 5%. Monomers with less than 5% mutual divergence are classified as the same monomer type, whereas those differing by more than 5% are considered distinct types. Over the past several decades, alpha satellite HORs in incomplete centromeric assemblies have been extensively investigated [5,6,7,8,9,10,11,12,13,14,15,16,17,18,19]. In particular, studies in the 1990s provided the first evidence of HOR structures in great apes, typically describing arrays of a few kilobases in size [20,21,22,23,24,25,26]. These pioneering works established the concept of alpha-satellite HORs in non-human primates, even though the approaches used at the time could not resolve the much larger superHOR structures described here.

To explore evolutionary dynamics across primate centromeres, fully assembled orthologous centromeres from chimpanzee, orangutan, and macaque genomes have recently become available. Comparative analysis revealed pronounced lineage-specific variations in the organization of alpha satellite HORs. A recent study using conventional HOR-detection algorithms showed that the centromeric architecture of the orangutan differs markedly from both human and chimpanzee, with alpha satellite HOR arrays organized into three to four distinct blocks, each sharing only 80–90% sequence identity. This mosaic-like arrangement suggests independent HOR expansions forming a patchwork pattern along the centromeric region [1].

In contrast, our application of the novel GRMhor algorithm (formerly GRM2023) led to the discovery of a 59mer superHOR, termed the cascading alpha satellite HOR, within the complete centromere of orangutan chromosome 13 [27]. This finding is particularly noteworthy, as the previously longest known canonical alpha satellite HOR was the 34mer located in the human Y chromosome. GRMhor allows for the identification of exceptionally large and structurally complex HOR units, including canonical HORs that feature internal repetitions of identical monomer types.

To further characterize these newly identified structures, we applied GRMhor to the complete assembly of the orangutan Y chromosome. Our analysis revealed a dominant 58mer cascading superHOR array, alongside a less pronounced 45mer HOR array, both exhibiting lower divergence and a more distinct structural organization than the canonical 34mer HOR found in the human Y centromere. These findings are detailed below using GRM and MD diagram representations.

## 2. Results

### 2.1. GRM and MD Diagrams for Orangutan Y Chromosome

**Figure 1:**
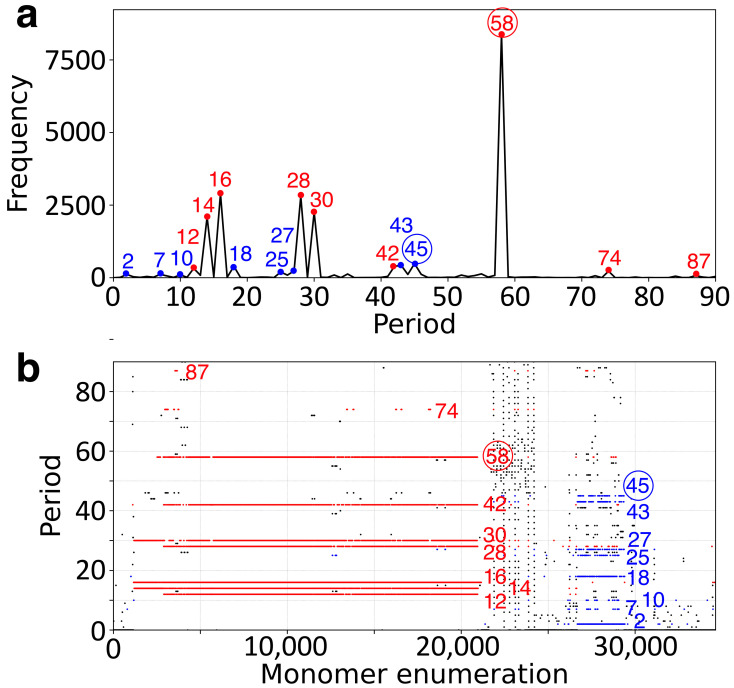
GRM (Global Repeat Map) diagram and MD (Monomer Distance) diagram for tandemly arranged alpha satellite monomers in the complete assembly of the orangutan Y chromosome. An MD-point of period *p* and monomer enumeration *l* represents a monomer with enumeration *l*, followed by the next occurrence of the same monomer type at a distance *p*. The MD diagram also includes several scattered points representing less frequent or random repeat patterns. For a detailed explanation of GRM and MD diagram construction, see Section 4 and [27]. (**a**) GRM diagram. The horizontal axis represents repeat periods (in units of ~171 bp), and the vertical axis indicates the frequency of those periods. The dominant peak at period 58 corresponds to the primary 58mer HOR (highlighted in red), while additional peaks at periods 42, 30, 28, 16, 14, and 12 (also in red) represent intra- and inter-58mer HOR copy subfragments—collectively referred to as the 58mer HOR family. A secondary peak at period 45 corresponds to the 45mer HOR (highlighted in blue), with associated peaks at similar lower periods (colored blue) representing its subfragments—collectively referred to as the 45mer HOR family. (**b**) MD diagram. The horizontal axis shows the enumeration of tandemly arranged alpha satellite monomers (~3500), while the vertical axis shows the repeat period (i.e., the distance between monomers of the same type). Horizontal lines from ~1000 to ~21,000 correspond to the 58mer HOR family (in red), and lines from ~26,500 to ~29,500 correspond to the 45mer HOR family (in blue).

As an initial step in our analysis, we applied the GRMhor algorithm to the complete assembly of the orangutan Y chromosome to generate the corresponding GRM and MD diagrams. Peaks observed in the GRM diagram indicate the presence of alpha satellite repeats, specifically corresponding to *n*mer HOR arrays, as well as intra- and inter-HOR copy repeat structures (Figure 1a). The most prominent GRM peak, at period 58, reflects the dominant 58mer HOR (highlighted in red circle), while another notable peak at period 45 corresponds to the 45mer HOR (highlighted in blue circle). Additional peaks at lower periods are associated with subfragments of the 58mer and 45mer HORs, and some peaks at higher periods arise from HOR structural variants.

The MD diagram (Figure 1b) reveals a dominant horizontal line segment at period 58, spanning the monomer enumeration range from approximately 1000 to 21,000. This pattern corresponds to the 58mer HOR array (highlighted in red). Within the same range, additional parallel but interrupted line segments are observed at periods 42, 30, 28, 16, 14, and 12, which reflect substructures within or between 58mer HOR copies (intra- or inter-HOR copy repeats).

A second, less distinct horizontal line segment appears at period 45, located in the enumeration range from approximately 26,500 to 29,500, and is associated with more divergent 45mer HOR copies (Figure 1b, blue). Below this, further interrupted lines appear at periods 43, 27, 25, 18, 10, 7, and 2—likely representing subfragments of the 45mer HOR.

**Figure 2:**
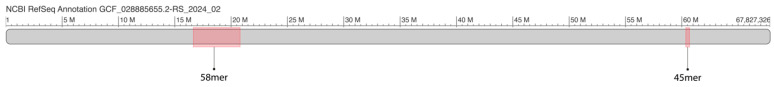
Ideogram of alpha satellite HOR arrays in the orangutan Y chromosome. The figure illustrates the chromosomal locations of the two major alpha satellite HOR arrays: the dominant cascading 58mer HOR array and the less prominent cascading 45mer HOR array. The 58mer HOR region spans monomer enumeration positions ~1000 to ~21,000, while the 45mer HOR region is located between ~26,500 and ~29,000 as indicated in the MD diagram (Figure 1b).

Notably, two additional short MD-line segments at periods 74 and 87, found above period 58, are associated with structural variants of the canonical 58mer HOR. The genomic positions of both the 58mer and 45mer HOR arrays are also depicted in the ideogram (Figure 2).

Novel cascading 58mer HOR alignment scheme in orangutan Y centromere

Based on the GRM and MD diagram analyses (Figure 1a,b), we identified 310 copies of the cascading 58mer alpha satellite HOR within the orangutan Y chromosome. Among them, 258 are canonical HOR copies, while the remaining 52 are structural variants (Table 1, Supplementary Table S1). The organization of the array follows a specific pattern of canonical (C) and variant (V) copies, represented as a linear sequence:

VCVVVCCCCCVVVVCVVVVVCCCCCCCCCCCVCCVCCCCCCCVVVVCCCVCCCCCCCCCCCCCCCCCCCCCCCCCCCCCCCCCCCCCCCCCCCCCCCCCCCCCCCCCCCCCCCCCCCCCCCCCCCCCCCCCCCCCCCCCCCCCCVCVCCCCCCCCCCCCCCCCVCCVVCCCVCCCVVVCVCCVVVCVCCCCCCVCCCCCCCCCCCCCCCCCVVCVCVVCCCCCCVCCCCVCCCCVCCCCCCCCCCCCCCCCCCCCCCCCCCVVVVCCCVCCCCCCCVCCCCCCVCCCCCVVCCCCCCCCCCCCCCCCCCC,

**Table 1:** Monomer counts in canonical and variant HOR copies of the 58mer array.

HOR Monomeric Scheme	No. of Monomers in 7 Successive Rows							No. of Monomeric Rows in HOR
	1	2	3	4	5	6	7	
C58	13	16	–	–	7	4	18	5
V74	13	16	16	–	7	4	18	6
V90	13	16	16	16	7	4	18	7
V28	–	–	–	–	6	4	18	3
V87	13	16	16	13	7	4	18	7
V59	14	16	–	–	7	4	18	5
V57	13	15	–	–	7	4	18	5
V55	13	16	–	–	7	4	15	5
V42	13	–	–	–	7	4	18	4

Each row indicates the number of monomers in up to seven aligned rows for each HOR copy. The canonical 58mer HOR (C58) contains five rows: 13, 16, –, –, 7, 4, and 18 monomers, respectively. Variants (e.g., V74, V90) differ by insertion or deletion of rows and/or monomers. Dashes (–) indicate missing rows. The final column shows the total number of rows present in each HOR copy.

**Figure 3:**
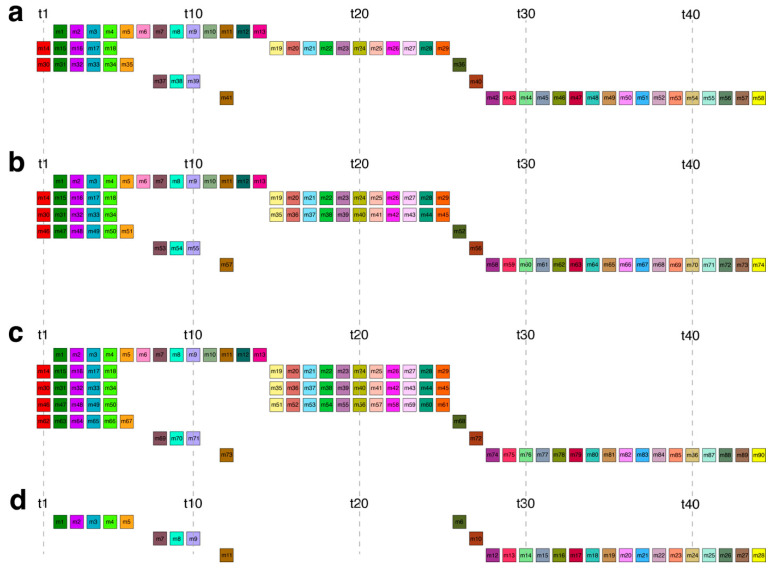
Schematic representation of aligned HOR monomeric structures within the cascading 58mer HOR array in the orangutan Y chromosome. (**a**) Canonical 58mer HOR copy. (**b**) 74mer HOR copy variant. (**c**), 90mer HOR copy variant. (**d**) 28mer HOR copy variant. Among the 58 monomers constituting the canonical HOR unit, 44 are of distinct types, labeled t1 through t44. Each monomer type is color-coded and shown as a distinct box in the schematic. As shown in the MD diagram (Figure 1b), the region from ~1000 to ~21,000 in monomer enumeration corresponds to the canonical 58mer HOR array (period 58). In the aligned HOR-monomeric scheme, monomers in each row are of different types, while those in each column are of the same type.

The complete alignment scheme of the 58mer HOR array is provided in Supplementary Figure S1, while the consensus sequence of the canonical 58mer HOR unit is shown in Supplementary Table S2. Representative monomeric structures for the canonical and the first three variant copies are schematically illustrated in Figure 3a–d. The average divergence among canonical 58mer HOR copies is extremely low (0.25%), whereas the 45mer HOR copies exhibit slightly higher divergence (0.81%).

The canonical 58mer HOR copy (Figure 3a) is organized into five monomeric rows containing 13, 16, 7, 4, and 18 monomers, respectively. In comparison, the 74mer variant (Figure 3b) includes six rows, with monomer counts of 13, 16, 16, 7, 4, and 18, indicating a duplication of the second row. The 28mer variant (Figure 3d) is composed of three rows with 6, 4, and 18 monomers, respectively.

Table 1 provides a comparative summary of the canonical and variant HOR copies in the 58mer HOR array, based on row alignment. This alignment reveals a high degree of columnar conservation across copies, with occasional shifts resulting from row insertions, deletions, or monomer-level changes. All observed variant structures can be derived from the canonical configuration through simple duplications or deletions of rows, or by insertion/deletion of individual monomers within rows. For instance, the 74mer variant emerges by repeating the second row of the canonical scheme as the third row, while the 28mer variant results from deleting the first and second rows and removing the first monomer from the fifth row.

### 2.2. Novel Cascading 45mer HOR

**Figure 4:**
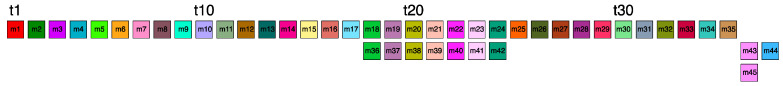
Schematic alignment of the canonical cascading 45mer HOR copy in the orangutan Y chromosome. The HOR unit is arranged into three monomeric rows, with 35 monomers in the first row, 9 in the second, and a single monomer in the third row. The presence of repeated monomer types across rows reflects a weakly cascading structure. Each monomer type is color-coded and represented as a distinct box in the schematic. Monomers in different rows correspond to different types, whereas monomers aligned in the same column belong to the same type.

The canonical cascading 45mer HOR identified in the orangutan Y chromosome consists of 45 monomers, of which only 37 are of distinct types (labeled t1 through t37). This HOR copy is organized into three rows (Figure 4): the first row contains 35 monomers, the second contains 9, and the third row consists of a single monomer. Due to its shallow multi-row structure, this configuration can be classified as weakly cascading.

In the MD diagram (Figure 1b), the canonical 45mer HOR is represented by the top horizontal line segment within the monomer enumeration range of approximately 26,500 to 29,500.

### 2.3. MD-Frequency Table for Orangutan Chromosome Y

For each MD period, the corresponding repeat frequency was calculated based on the complete centromeric assembly of the orangutan Y chromosome (Table 2). The highest observed frequency, 8389, corresponds to the canonical 58mer HOR. Several additional peaks, with frequencies of 391, 2254, 2803, 2891, 2089, and 322, represent subfragments within or between 58mer HOR copies.

Among the remaining frequencies, the next highest peak (frequency 484) corresponds to the 45mer HOR (Figure 4), accompanied by a series of lower-frequency peaks (43, 35, 27, 25, 18, 10, 7, and 2) reflecting its subfragments. Notably, period 74 (frequency 225) aligns with the most prominent variant of the 58mer HOR (Figure 3b), while the only higher-order variant with a frequency above 60 is at period 87 (frequency 74), corresponding to the second most prominent 58mer variant (Figure 3c).

### 2.4. Cascading 18mer HOR in Gorilla and Willard’s Type 28mer HOR in Chimpanzee Chromosome Y

**Figure 5:**
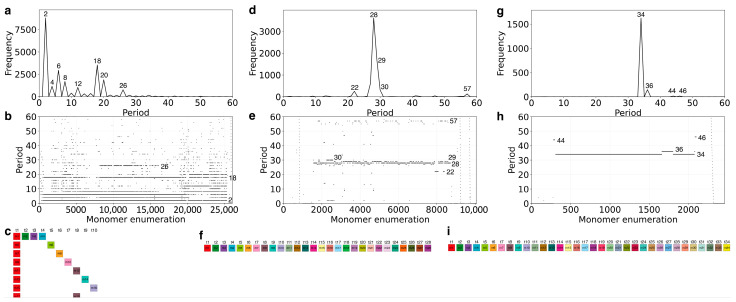
GRM (Global Repeat Map) and MD (Monomer Distance) diagrams, along with schematic monomeric organization of alpha satellite HORs, in complete Y chromosome assemblies of great apes and humans. (**a**–**c**) Gorilla: The GRM and MD diagrams (**a**,**b**) reveal a dominant 18mer cascading HOR, characterized by frequent binary monomer pairings. The corresponding monomeric organization (**c**) shows repeated doublet structures, contributing to elevated subfragment frequencies (e.g., period 2). (**d**–**f**) Chimpanzee: A canonical 28mer HOR of Willard’s type is identified (**d**,**e**), where each monomer is unique within the repeat unit. The monomeric scheme (**f**) reflects this non-redundant structure. (**g**–**i**) Human (T2T-CHM13): The GRM and MD diagrams (**g**,**h**) show a dominant 34mer Willard-type HOR, along with minor 36mer variants. The monomeric alignment (**i**) confirms the non-repetitive, single-copy monomer structure of the canonical HOR.

To extend our comparative analysis, we applied the GRM2023 algorithm to the complete Y chromosome assemblies of gorilla (GCF_029281585.2, NHGRI_mGorGor1-v2.0_pri) and chimpanzee (GCF_028858775.2, NHGRI_mPanTro3-v2.0_pri). The resulting GRM and MD diagrams, along with the monomeric organization of the respective HORs, are shown in Figure 5a–f. For reference, results from the human Y centromere (GCF_009914755.1, T2T-CHM13v2.0) are presented in Figure 5g–i.

In the gorilla Y chromosome, the dominant MD-line segment (Figure 5b) corresponds to a repeat period of 18, identifying the canonical 18mer HOR (Figure 5c). This HOR exhibits a distinctly cascading organization, composed primarily of repeating monomer doublets (t1 t*n*). Due to this binary repetition pattern across HOR copies, the period-2 subfragment (MD-frequency 8814) exceeds even the frequency of the full 18mer repeat (3542), as shown in Figure 5a.

In the chimpanzee Y chromosome, the highest MD-frequency (3613; Figure 5d) corresponds to a 28mer HOR (Figure 5e), which exhibits the classical Willard-type architecture—each monomer appears only once within the HOR unit (Figure 5f).

For comparison, the human Y centromere shows a dominant 34mer HOR with a maximum MD-frequency of 1626 (Figure 5g), and a smaller number of variant 36mer HORs (frequency 148). The canonical HOR also conforms to the Willard-type organization (Figure 5h,i), consistent with earlier studies based on partial sequencing data [7].

### 2.5. Comparison of Alpha Satellite HORs in Human Y Chromosome Assemblies GCA_018873775.2 and T2T_CHM13v2.0

To assess whether the relatively low abundance of alpha satellite sequences in the human Y chromosome, compared to the orangutan genome, could be attributed to assembly methodology, we examined the genome assembly GCA_018873775.2 from the Human Pangenome Reference Consortium, which includes a complete Y chromosome.

**Figure 6:**
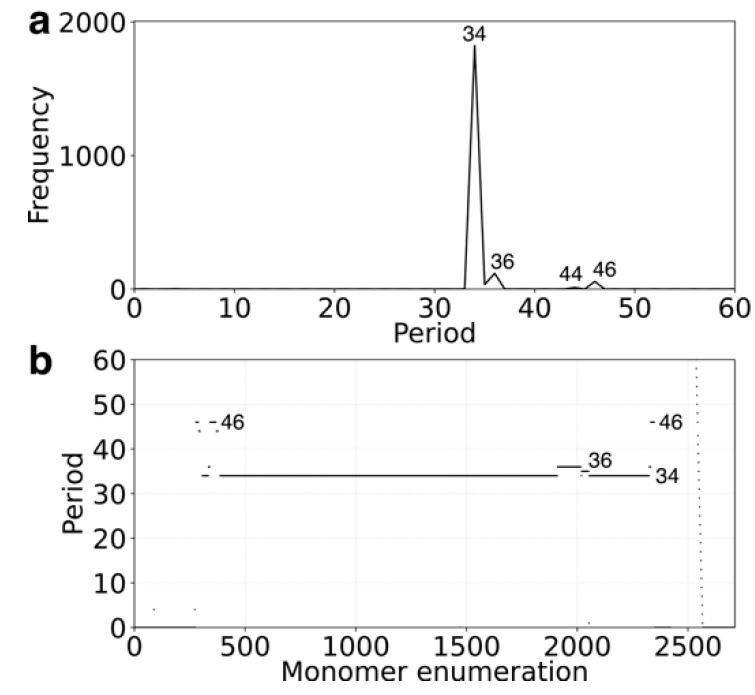
GRM (Global Repeat Map) and MD (Monomer Distance) diagrams for alpha satellite monomers in the complete Y chromosome assembly of GCA_018873775.2. (**a**) GRM diagram. The *x*-axis represents repeat periods (in ~171 bp monomer units), and the *y*-axis shows their frequency. A dominant peak is observed at period 34, corresponding to the canonical 34mer HOR. Additional peaks at periods 36, 44, and 46 reflect structural variants and subfragments within and between HOR copies. (**b**) MD diagram. The *x*-axis indicates the sequential enumeration of alpha satellite monomers (~2500 total), while the *y*-axis represents the period distance between repeated monomer types. The diagram confirms a highly regular 34mer HOR structure, with variant units (e.g., 46mer and 36mer) appearing at the beginning and end of the array.

As shown in the GRM and MD diagrams (Figure 6a,b), the alpha satellite organization in GCA_018873775.2 is largely consistent with that of the T2T-CHM13v2.0 assembly. Although GCA_018873775.2 contains approximately 10% more alpha satellite sequence, this additional content still conforms to the same canonical 34mer HOR structure.

Notably, in contrast to T2T-CHM13v2.0, the HOR array in GCA_018873775.2 begins with a 46mer HOR (rather than the 44mer variant) and includes a duplicated copy in the third HOR unit. At the array’s end, a third structural variant appears as a 36mer HOR (Figure 6b). Overall, the extra alpha satellite content slightly extends the length of the 34mer HOR array in GCA_018873775.2, while preserving its core organization.

## 3. Discussion

### 3.1. Comparative Overview of Y-Centromere HOR Architectures

Our comprehensive analysis of alpha-satellite HORs across complete Y-chromosome assemblies in human and non-human great apes reveals substantial interspecies differences in HOR organization, despite low divergence within canonical HOR copies in each species (Table 3). Humans and chimpanzees both exhibit Willard-type HORs, in which each monomer within the HOR unit is unique. In contrast, orangutans and gorillas possess more complex cascading-type HORs, characterized by internal repetition of monomer types and multi-row architectures.

A particularly striking feature is the discovery of a massive 58mer cascading superHOR in the orangutan Y centromere—significantly larger and more structured than the canonical 34mer HOR in human Y. While previous studies described the orangutan centromere as a “mosaic patchwork” of partially diverged HOR blocks [1], the application of our GRMhor algorithm revealed a highly ordered array with minimal divergence (0.25%) and consistent monomeric structure. We also identified a secondary 45mer HOR and additional superHORs in other orangutan chromosomes, indicating that long, stable HOR arrays may be a recurring architectural feature of this species’ centromeres.

Among the examined species, gorilla centromeres exhibit an unusual pattern: the frequency of inter-HOR subfragments (e.g., period-2 repeats) exceeds that of the full HOR unit, suggesting unique structural dynamics or recent homogenization processes. These findings support the notion that alpha satellite HOR architectures are both species-specific and evolutionarily dynamic, providing a framework for understanding genome evolution at centromeric loci.

It is important to note that the absence of very large, highly ordered “superHOR” structures in the current gorilla and chimpanzee Y chromosome assemblies reflects genuine biological absence rather than detection failure, as these assemblies are now complete and gapless in the centromeric regions. In earlier reference genomes for these species, which relied primarily on short-read sequencing, large repetitive structures could not be fully resolved and might have been overlooked. In contrast, the present study uses complete assemblies for all examined species together with the GRMhor algorithm, ensuring that superHORs on the Y chromosome would be detected if present. While no such superHORs are found in gorilla or chimpanzee Y centromeres, the possibility remains that analogous structures could exist elsewhere in their genomes, outside the Y chromosome, and this will require targeted investigation in future work.

### 3.2. Observations Versus Interpretations

The exceptionally low divergence and highly regular columnar organization of the orangutan 58-mer and 45-mer superHORs are direct observations from our analyses; by contrast, any proposed effects on kinetochore robustness or higher-order chromatin architecture remain hypotheses that will require targeted functional validation.

### 3.3. Functional Implications for Kinetochore and Chromatin

Large canonical HOR arrays with extremely low divergence, such as the 58mer superHOR described here (0.25% mean divergence), may provide a particularly stable and homogeneous DNA substrate for kinetochore assembly. Centromere function depends on the precise recruitment of centromere-specific proteins, most notably CENP-A, which replaces histone H3 in a subset of nucleosomes and defines the epigenetic centromere location [16,28]. Highly regular HOR structure could facilitate efficient and reproducible CENP-A nucleosome positioning, potentially enhancing kinetochore robustness.

The internal periodicity and repeat homogeneity of such arrays may also influence higher-order chromatin folding. Alpha satellite DNA has been shown to adopt specific nucleosome phasing patterns [18] and to interact with CENP-B and other DNA-binding proteins that recognize conserved motifs within monomers [29]. The cascading organization we observe—where identical monomer types are repeated within the canonical HOR—could provide multiple, regularly spaced protein-binding sites within a single HOR unit, possibly increasing cooperative binding and stabilizing local chromatin loops.

From an evolutionary perspective, large HOR arrays may impact meiotic stability. Extended arrays of near-identical repeats are substrates for non-allelic homologous recombination (NAHR), which can lead to expansions, contractions, or rearrangements [30]. While such changes could drive evolutionary novelty, they may also pose a risk of segregation errors if array integrity is disrupted. The structural integrity of the orangutan Y 58mer superHOR suggests strong selective pressure to maintain this architecture.

Taken together, these observations support the idea that large, highly ordered HOR arrays are not merely neutral repeat expansions but may be evolutionarily selected features contributing to kinetochore efficiency, chromatin architecture, and faithful chromosome segregation during both mitosis and meiosis. Future functional studies—particularly CENP-A ChIP-seq mapping in orangutan—would help to test these hypotheses directly.

### 3.4. Evolutionary Dynamics and Concerted Evolution

The extremely low divergence among canonical HOR copies in the orangutan Y centromere (0.25% for the 58mer, 0.81% for the 45mer) suggests a recent or ongoing homogenization process. Concerted evolution, driven by mechanisms such as unequal crossing-over and gene conversion, is known to maintain sequence uniformity across tandemly repeated DNA arrays [9,31]. In centromeric alpha satellite DNA, such processes can act over long evolutionary timescales to replace older, divergent repeat units with newer, homogenized copies, often resulting in long stretches of highly regular HOR structure. The size and homogeneity of the orangutan 58mer superHOR—spanning ~20,000 monomers with almost perfect columnar alignment—are consistent with a scenario in which a recent expansion of a single HOR haplotype has swept through the array. Alternatively, a combination of selective constraints on kinetochore function and ongoing homogenization could be maintaining this exceptionally ordered state. Comparative analysis of orthologous loci in multiple orangutan individuals, and in closely related species such as the Bornean and Sumatran orangutans, may help to distinguish between a single recent homogenization event and continuous turnover dynamics.

### 3.5. Linking Alpha-Satellite HOR Architecture to NBPF HORs and Functional Context

The organizational logic we uncover in centromeric alpha-satellite HORs—large, highly ordered arrays with exceptionally low divergence—parallels HOR structuring observed in non-centromeric gene families. In our prior work on NBPF (Olduvai) repeats [14,32,33], we showed that humans harbor ~50 tandemly organized NBPF 3-mer HORs that are absent in chimpanzees, pointing to an evolutionary shift from “more monomers” to “more organized monomers” (i.e., higher-order structure) as a potential mechanism for collective, synergistic effects at the locus level. This echoes the idea that it is the HOR organization—rather than copy number alone—that can drive coherent genomic or chromatin outcomes.

Methodologically, the same GRMhor framework used here was developed to resolve both classical (Willard-type) and cascading HORs, and has been applied successfully to short NBPF 3-mer HORs as well as long alpha-satellite HORs. This cross-context capability supports a unified view in which HOR architecture—whether in centromeric satellites or in multigene families—encodes higher-order regularity that can impact local chromatin folding, protein binding site periodicity, and the stability of long tandem arrays.

In centromeres, such regularity has obvious implications for kinetochore assembly and epigenetic specification (e.g., positioning of CENP-A nucleosomes); by analogy, repeating phased NBPF HOR modules may favor cooperative interactions (e.g., nucleosome phasing or motif periodicity) that scale from monomer to array. While direct functional testing remains beyond the scope of this study, the convergence of HOR principles across centromeric alpha satellites and NBPF arrays strengthens the broader claim that higher-order structuring is an evolutionarily selected, information-bearing feature of tandem DNA, not merely a by-product of repetition.

### 3.6. Limitations

While the GRMhor algorithm is highly sensitive in detecting both classical (Willard-type) and cascading HORs, certain limitations should be acknowledged. First, in regions with extreme local sequence divergence or structural rearrangements, alignment noise may occasionally obscure the precise boundaries of HOR units, potentially leading to borderline cases in canonical vs. variant classification. Second, while our <5% divergence threshold for monomer type assignment is consistent with the literature, sequence artifacts (e.g., residual assembly errors, base-calling inaccuracies in long-read data) could, in rare cases, result in false splitting or merging of monomer types. Third, for HOR arrays with extensive internal duplications and multiple variant subfamilies, there is an inherent risk that complex rearrangements could mimic cascading structures. To mitigate these risks, we applied GRMhor exclusively to complete, gapless assemblies and visually confirmed all HOR classifications through aligned schematic representations and cross-validation with GRM and MD diagrams. Nevertheless, as with any computational annotation of highly repetitive DNA, a small probability of classification ambiguity remains.

## 4. Materials and Methods

The analyses presented in this study were performed using the GRMhor (Global Repeat Map HOR) algorithm, a recently developed method for the comprehensive identification, classification, and visualization of HOR structures within tandemly repeated monomer arrays. A full technical description of GRMhor, including its core components, performance benchmarks, and comparisons with other existing tools, is available in our recent publication [34].

### Step-by-Step GRMhor Pipeline

All analyses were performed using the GRMhor algorithm [34] in combination with the MonFinder tool.

1. Monomer extraction—MonFinder identifies alpha satellite monomers by aligning the target genomic sequence to a 171 bp consensus alpha satellite monomer, searching in both forward and reverse complement orientations. Matches with ≥95% identity are retained as monomers.
2. Monomer type classification—Each monomer is assigned to a specific monomer type if its sequence differs by less than 5% from other members of that type. Once monomer types are determined, the structure of the HOR array is established by vertically aligning monomers of the same type (i.e., those mutually differing by <5%) into columns. This process produces the aligned HOR schematic, in which each column contains monomers of a single type positioned consistently across HOR copies. Such vertical alignment not only enables a clear visual representation of the HOR structure but also ensures that monomer type assignment is consistent with the positional architecture of the array. This approach improves robustness against misclassification that could arise if only pairwise divergence was considered, by integrating sequence similarity with structural context in the final HOR diagram.
3. Treatment of gaps—In the general case, undefined bases (‘N’) in the genomic assembly are ignored. Any monomer overlapping such a region is excluded from analysis to prevent erroneous HOR detection. In this study, however, all organisms were analyzed using complete assemblies without gaps in the targeted centromeric regions (orangutan, human, chimpanzee, gorilla), so this filtering step was not required.
4. GRMhor analysis—The monomer list is analyzed to compute the GRM diagram (repeat periods vs. frequency) and the MD diagram (monomer positions vs. repeat period), using default parameters (max period = 90). No additional smoothing or pre-filtering was applied.
5. Canonical and variant HOR definition—Within each detected HOR array, the canonical HOR is defined as the most frequent, complete *n*-mer unit. HOR copies that deviate from this dominant structure—through insertions, deletions, or internal duplications of one or more monomers—are classified as variant HORs. GRMhor assigns these labels by aligning all HOR copies monomer-by-monomer to the canonical reference and quantifying structural concordance; variant types are explicitly annotated in the aligned HOR schemes presented in the Results. This definition ensures that structural comparisons are made relative to a clearly established reference within each array.
6. Output—For each HOR family, GRMhor outputs graphical representations (GRM, MD, aligned HOR scheme) and tabulated monomer composition, enabling full reconstruction of HOR organization.

Both MonFinder and GRMhor are freely available at: https://github.com/gluncic/GRM2023 (accessed on 15 August 2025).

In this study, we applied GRMhor to the complete Y chromosome assemblies of the orangutan, human, chimpanzee, and gorilla, using the following genomic datasets:

- Orangutan (NHGRI_mPonAbe1-v2.0_pri, GCF_028885655.2, National Human Genome Research Institute, National Institutes of Health, 5 January 2024)
- Human (T2T-CHM13v2.0, GCF_009914755.1, T2T Consortium, 24 January 2022) and (HG01243v3.0, GCA_018873775.2, Johns Hopkins University, 27 September 2021)
- Chimpanzee (NHGRI_mPanTro3-v2.0_pri, GCF_028858775.2, National Human Genome Research Institute, National Institutes of Health, 8 January 2024)
- Gorilla (NHGRI_mGorGor1-v2.0_pri, GCF_029281585.2, National Human Genome Research Institute, National Institutes of Health, 8 January 2024)

Importantly, GRMhor enables detection of not only traditional Willard-type HORs, where each monomer in a unit is unique, but also more complex-type HORs, characterized by repeated monomer types within the same unit—such as the 58mer and 45mer superHORs described in this study. This capability is crucial for identifying previously undetectable structures in complex regions like the Y chromosome centromere.

## Figures and Tables

**Table 2:** MD frequencies in the orangutan Y centromere.

Period	2	7	10	12	14	16	18	25	27	28	30	33	35	42	43	44	45	46	52	55	58	74
Freq.	113	118	97	322	2089	2891	393	197	233	2803	2254	94	128	391	428	114	484	117	91	132	8389	225

Frequencies represent the number of MD-points for each period, calculated from the complete alpha satellite monomer array in the orangutan Y centromere. Period 58 shows the highest frequency (8389), corresponding to the canonical 58mer HOR. Red and blue shading indicates periods and subfragments associated with the 58mer and 45mer HOR families, respectively. Only periods with frequencies >90 are shown.

**Table 3:** Dominant HOR arrays in great ape Y chromosomes.

	Human	Chimpanzee	Gorilla	Orangutan
*n*mer HOR	34mer	28mer	18mer	58mer
No. HOR copies	54	259	779	310
No. Canonical HOR copies	49	237	405	258
HOR-type	Willard’s	Willard’s	Cascading	Cascading

Summary of canonical alpha satellite HOR arrays identified in complete Y chromosomes of human, chimpanzee, gorilla, and orangutan assemblies. Human and chimpanzee exhibit Willard’s-type HORs (each monomer type appears only once per HOR unit), while gorilla and orangutan show Cascading-type HORs with repeated monomer types. The table lists the HOR unit length (nmer), total and canonical copy counts, and HOR type.

## Data Availability

Genomic sequence are freely available at the National Center for Biotechnology Information (NCBI) website https://www.ncbi.nlm.nih.gov (accessed on 15 August 2025). Both MonFinder and GRMhor are freely available at: https://github.com/gluncic/GRM2023 (accessed on 15 August 2025).

## Supplementary Materials

The supporting information can be downloaded at: https://www.mdpi.com/article/10.3390/ijms26178122/s1.
