# Supplementary material for: Cascading 58mer Alpha Satellite superHOR in Complete Orangutan Y Chromosome
Source: Int J Mol Sci. 2025 Aug 22;26(17):8122. doi: 10.3390/ijms26178122 (PMC12428378; doi:10.3390/ijms26178122)
Supplement: Supplementary file 1 [file ijms-26-08122-s001.zip › supplementary_materials.pdf]

Supplementary Materials for

Cascading 58mer alpha satellite superHOR in complete orangutan Y chromosome

Matko Glunčić, Ines Vlahović, Marija Rosandić, Vladimir Paar

Corresponding author: matko@phy.hr

The Supplementary file includes:

Fig. S1
Tables S1, S2

Fig. S1 (separate file) Cascading 58mer alpha satellite HOR alignment. Start position 18,488,201 bp and end position 21,586,501 bp in NHGRI_mPonAbe1-v2.0 chromosome Y. The number on the left side indicate the initial position of the first monomer in each row of HOR copy.

Table S1. Canonical and variant HOR copies in the orangutan 58mer HOR array

HOR	copy Number	%
Canonical 58mer	46	68,7
Variant 74mer	5	7,5
Variant 90mer	2	3,0
Variant 28mer	2	3,0
Variant 87mer	2	3,0
Variant 59mer	1	1,5
Variant 57mer	3	4,5
Variant 55mer	2	3,0
Variant 42mer	4	6,0
Total	67	100

Table S2. Canonical 58mer consensus sequence.

Monomer type t1
TCAGAAACTTCTTTGTGATGAGTGCCTTCAACTCACAGAGTTGAACGTTCCTTTTGATAGAA
CAGTTTTGAAACACTGTTTTGGTAGTATCTGGAAGTGTTGATTCGGAGCGCTTTGAGGCCTG
TGTTGGAAAAGGAAATATCTTCACGTAAAAAGTAGACAGAAGCATTC
Monomer type t2
TCAGAAACTTCTTTGTGAGGAGTGCATTCAACTCACAGAGTTGAACCTTCCTATTGATAGAG
AAGTTTTGAAACACCCTTTTTGTAGAATTTGCAGGTGAATATTTGGAGCGATTGGAGACCTA
TGCTTGAAATGGAAACATCTTCACATGAAAACTAGACGGAAGAACTC
Monomer type t3
TCAGAAACCTCTTCGTAATGAGTGCTTTCAACTCACAGAGTTGAAGCTTCCTTTCCAAAGAG
CAGTTTTGAAACACTTTTCTGTAGAATCTGGAAGTGGATATTTGGAGCGCTTTGAGGCCTAT
GCTGGGAAGTGAAATATCTTCACCTAAACACTAGACTGAAGCATTC
Monomer type t4
TCAGAAACTTCTTCACGATCTGTGTATTCAACTCACAGCTTTGAACCTTTCTTTTCGTAGAG
CAGTTTTGAAACACACTTTTTGTAGAATCTGCAACGGCATATTTGGAGCGCTTTGAGGCGTA
TGCTTGAAAAGGAAATATTTTCACATAAACGCTAGACAGAAGCATTC
Monomer type t5
TCAGAAACTTCTTTCTGATGTGTGCATTCAACACACAGAGTTGAACCTGCCTTTTGATAGAG
GAGTTTGGAAACACCCTTTTTGTAAAATCTGCAAGTTGATATTTGGAGCTATTTGAGGCCTA
TGCTGTAAAAGAAAATATTTTCACATAAACACTAGACAGAAGACGTC
Monomer type t6
TAAGAAACTTCTGTGTGGTGAGTGCATTCAGCTCATAGGTTTGAACGTTGGTTTTGATAGAA
CATTTTGGAAAGACCCCTTTTGTAGAATCTGCAGATGTTTATTTGGAGCGCTTTGAGGCCTA
TGTTGGAAAAGGAAATTTCTTCACATAAAAACTAGACAGAAGCATTC
Monomer type t7
TCAGAAACTTCCTTGTGATGAGAGCATTCTACTCACAGAGTTGAACGTTCCTTTTGATAGAA
CAGTTTTGAAACACTCTTTTTAGAGACTCTGTAAGTGGCTACTTGGAGCGCTTTGAGTCCTA
TGCTGGAAAACGAAATATCTTCACATAAAAACAACAGCGAAGCACTC
Monomer type t8
TCAGAAACATTTTGCGAGGTGCGCATTCAACTCACCGAGTTGAACCTTCCTTTTGGTAGAGC
AGTTTTGAAACACTCTTTTTCTAGGATCTGCAAGTGGATATTTGGAGTGCTAAGAGGCCAAC
ACTGGAAAAGGAAATAACTTCACATGAGAACTAGACGGAAGCATTG
Monomer type t9
TCAGAAACTTCCGTGTGCTTTATGCCTTCAACTCAAATAGTGGAATCTTCCTTTTGATAGCA
CAGTTTTGAAACACTCCTTTTGTAGAATTTGCTAGTGGATGTTTGGAGCGATTTGAGGCCTA
TGCTGGAAAAGGAAATATCTTCACATAAAAACTAGAGAGAAGCGTTC
Monomer type t10
TCAGAAACTTCTTTATGATGAGTGCATTCAACTCACAGACTTGAACCTTCCTTTTGGAAGAG
CAGTTTTGAAACACTCTTTTTGTAGAATCTGCAAGTGGATATTTAGAGCGCTTTGAAGCCTA
TGCTGGGAAATGAAATATCTTCACCTAAAACGTAGACAGTAGCATTC
Monomer type t11
TCTGAAGCTTCTTCACGATGCGTGCATTCAACTCACAGAGTTGAATCTCATTTTGATAGAGC
AGTTTGGAAACACTCTTTTTGTAAAATCTGCAAGTGGATATTACGAGGGCTTCGGGTCAATG
TTGGAAAAGGAAATATCTTCACATGAAAACTAGACAGAAGCATTC
Monomer type t12
TCAGAAACTTCTTGGTGATGAGTGCATTCAACTCACAGACTTGAACCTTCCTTTACAAAGAG
CAGTTTAGAAACACAGTTTTTGTAGAATCTGAAAGTGGATATTTGGAGCGCTTTGAGGCGAG
GCTGGGAAATGAAATATCTTCACCTAAAAACTAGACAGAAGCATTC
Monomer type t13

TCAGAGACTTCTTCCTCATGTGCGCATTCAACTCACAGAGTTGAACCTGCCGTTTCACAGAG
CAGTTTGGAAACACTCTATTGGTGTAAACTGCAATTCGATATTTGGAGCGATTTGAGGCCTA
TGCAAGGAAAAGGAAATATCTTCTGATAAAAACTAGACTGAATCATTC

Monomer type t14
TCAGAACCTTCTGTGTGATGAGAGCATTCAATTCACAGACTTGAACCTTCCTTCTGATAGAG
CACTTTTGAGACCCACTTTCATTGCCATCTGAAGATGGATGTTTGGAGCGCTTTTTGGCCAA
TGCTGCAATACGAAATATCATCACATAAAAACTAGACAGAAGCATTC

Monomer type t15
TAAGAAACTTCTGTGTGATGAGTGCATTCAGCTCACAGACTTGAACGCTGGTTTTGATAGAA
CAGTTTGGAAACACCCCTCTTGTAGGATCTGCAGGTGTTTATTTGGAGCGCATTGAGGCCTA
GGTTGGAAAAGGAAATATCTTCACATAAACAGTAGACAGAAGCATTC

Monomer type t16
TCGGAAACTTCCTTGTGATGAGTGCATTGTACTCACAGAGTTGAAAGTTCCTTTTGATAGAA
GAGTTTTGAAACACTCTTTTTATAGACTCTACAAGTGGCTATTTGGAGCGCTTTGAAGCCCA
GGCGGGAAAACGAAATATCTTCACATAAGAACTAGACAGTAGCGTTC

Monomer type t17
TCAGAAACATTTTGCGAGGTGCGCATTCAACTCACCGAGTTGAAGCTTCCTTTTGGTAGAGC
AGTTTTGAAAAACTCTTTTTCTAGGATCTGCAAGTGGATATTTGGAGTGCTAAGAGGCCAGC
ACTGGAAAAGGAAATAACTTCACATGAGAACTAGACGGAAGCATTG

Monomer type t18
TCAGAAACTTCCGTGTGCTTCATGCATTCAACTCACATAGTGGAATCTTCCTTTTGATAGCA
CAGTTTTGACACACTCCTTTCGTAGAATTTGCTCGTGGATGTTTGGAGCGATTTGAGGCCTA
TGCTGGAAAAGAAAATATCTTCCCATAAAAACTAGACAGAAGAATTC

Monomer type t19
TCAGAAATCTTTTCCGATGCGTGCATTCAACTCACCGAGTGGAACCTGCGTTTTGATAGAGC
AGTTTTGAAACACTCTTTTTGTAGAATCTGAAAGTGGATATTTGGAGTGCTAAAGGGCCAAC
ACTGGAAAAGTAAATATCTTCACCTGAGAACTAGACGGAAGGATTC

Monomer type t20
TCAGAAACTTCCGTGTGCTTTATGCGTTCAACTCATATAGTGGAATCTTCCTTTGGATAGCA
CAGGTTTGAAACACTCCTTTTGTAGAATTTGCTACTGGATGTTTGGAGCGATTTGAGGCCTG
TGCTGGAAAAGGAAATACCTTCACCTAAAAAGTACACACAAGCATTC

Monomer type t21
TCAGAAACTGTTTCACCATACGTGCCTTCAACTCACAGAGTTGAAACTGCCTTTCGATAGGG
CAGTTTGGAAACACTCTCTTTGTAATATCTGCAAGGGGATATTTGGAGCGATTTGAGGCCTA
TGCAAGGAAAAGGAAATATCTTCGCCTAAAAACGAGACAGAAGCATTC

Monomer type t22
TCAGAAGCTTCTGTGGATGAGTGCATTCAACTCACAGACTTGAACCTTCCTTTTGATAGTTC
AGTTTTGAAACACTCTTTCAGTCCCATCTGAAGGTGGATATTTGGAGCACCTTTAGGCCAAT
GCTGGAAAATGAAATATCATCACATAAAAACTAAACAGAAGAATTC

Monomer type t23
TCAGAAACCTCTTTGTGATGAGTGCATTCAACTCACAGACGTGAAAGTTCCTGTAGATGGAA
GAGCTTTGAAACACTCTTTTGGTGGGATCTACAAGTGTTTATTCGGAGCACTTTGAGGCCTA
TGATGGAAAAGGAAATATCTTCACGTAGAAATTAGACAAAAGCATTC

Monomer type t24
TCAGCAAGTTCCCTGTGTTTTGGGCATTCGACACACAGAGTTCAACCTGCCTTTTGATAGAG
AAGTTTTGCAACACTCTTTTTGTACAATCGGCAAGTGGATATTCAGAGCGCTTTGAGGCCTA
TGTTGGAAAAGGAACTATCTTCACGTGGAAACTAGACAAAAGCATTC

Monomer type t25
TCAGAAAGTTCCCTGTGTTTTGGGCATTCAACACACAGAGTTCAACCTGCCTTTTGATAGAG
AAGTTTTGCAACACTCTTTTTGTGGAATCTGCAAGTGGATATTTGGAGAGCTTTAAGTCCTA
TGCTGCAAAAGGAAATCTCATTGCATACAAGATTGACAGAAGCATTC

Monomer type t26
TCAGAAACTTCCTTGTGATGAGTGCATTCTACTCACAGAGTTGAACGTTCCTTTCGATAGAA
CAGTTTTGAAACACTCTTTTTATAGACTCTGCAAGTGGCTATTTAGAGCGCTTTGAGTACTG
TGCTGGAAAACGAAATATCTTCACATAAAAACTAAACCGAAGCACTC
Monomer type t27
TCAGAAGCTTCTTCACGATGCGTGCATTTGACTCACAGAGTTGAACCTGCCTTTTGATAGAG
CAGTTTGGAAACACTCTTTTTGTAAAATCTGCAAGTGGATGTTAGGAGGGCTTCGGGGCCAA
TGTTGGAAAAGGAAATATCTTCACGTGAAAACTAGGCAGAAGCATTC
Monomer type t28
TCAGAGACTTCTTCATGATGTGTTCATTCAACTCACAGAGTTGATCCTGCCGTTTGACAGAG
CAGTTTGGAAATACTCTATTCGTATAATCTGCAAGTCGATATTTGGAGCGATTTCAGGCCTA
TGCAAGGAAATGGAAATATCTTCTGATAAAAACTAGACTGAAGTATTC
Monomer type t29
TCAGAACCTTCTGTGTGATGAGAGCATTCAACTCACAGACTTGAACCTTCCTTCTGATAGAG
CATTTTTGAGACCCTCTTTCAGTGCCATCTGAAGGCGGATATTTGGAGCGCTTTTTGGCCAA
TGTTGGAATAAGAAATATCATCCCATAAAAACTAGACAGAAGCATTC
Monomer type t30
TCAGAAACCTCTTTGTGATGAGTGCCTTCAACTCACAGAGTTGAACGTTCCTTTTGATAGAA
CAGTTTTGAAAAACTGTTTTTTTAGAATCTGCAAGTGTTTTTTGGAGCGCTTTGAGGCCTAT
GTTGGAAAAGGAAATGTCTTCACATACAAAGTAGACAGAAGCAATC
Monomer type t31
TCGGAATCTTCTTTGTGGTGAGTGCATTCAATTTACAGAGTTGAACCTTCCAATTGATAGAG
AAGATTTGAAACAGCCTTTTTGTAGAATGTGCAAGTGAATATTTGGAGGTATTTGAGGCCTA
GATTTTAAATGGAAGCATCTTCACATGAAAACTAGACAGGAGAATTC
Monomer type t32
TCAGAAACGTCTTTGTGATGAGTGCATTCAACTCACAGACTTGAAGCTTCCTTTTGAAAGAG
CAGTGTTGATACACGCTTTGCTAGAATGTGCAAGTGGATATATGGAGCGCTTTGAGGCCTAT
GCTGGAAAAGGATATCTCTTCACGTAAAAGCTGGAAAGAAGCATTC
Monomer type t33
TCAGAAACTTCTTTGTGATGTGTGCCTTGAACTCACAAAGTTCAACCTTTCTTTTGGTAGAG
AAGTTTTGAAACACTCTTTTAGTAGAATTTTCAAGTGGATGGATGGAGTGCTTGGAGGCCAA
TGCTGGAAAAGAAAATATGTTCACGTAGAAACTACATAAAAGCATTC
Monomer type t34
TCAGAAGGTTCCCTGTGCTTTGGGCATTGAACTCACAGAGTTCAACCTACCTTTTGATAGAG
CAGTTTTGAGACACTCTTCGTAGAATCTGCCAGTGGACATTAAGAGCGCTTTGAAGCCTTTG
CTGCAAAAGGAAATATCATTGCGTAAAAGCTAGATGGAAGCATTC
Monomer type t35
TGTGAACCTTCTGTGTGATGAGTGCATTGAACTCACAGAGTTGTACCTTCCTTTTGGTAGAA
AAGTATTGCAACACTGTTTTTGTAGAATTCGCAAGTGGATATTTGGAGCTCTTTGAGGCTTA
TGCTGGAAAAGGAAATATCTTCATATAGAAGCTAGACAGAAACATTA
Monomer type t36
TCAGAAACTTCTATGTCTTGAGGGGATTGAACTGAGAGAGTTGAAGATTCCTTTTCCTGGAG
CAGTCCTGCAACACTCTTTTTGTGGAAACTGCAGGTGGAAATTTGGTGCGATTGCGGCATCT
AGTGGAAAAGGAAATACTTGCAGATGAAAACTAGACAGAACCATTA
Monomer type t37
TCAGAAACCTCTTTGTGATGAGTGCATTCAACTCACAGAGTTGAACGTTCCTTTTGAAAGAG
CAGTTTTGAAACACTCTTTTTGTAGAATCTGCAAGTGGATATTTGGAGCGCTTTGAGGCCTA
TGCTGGGAAAGGAAATATCTTCACCTAAAAACTAGACAGAAGCATTC
Monomer type t38

TCAGAAACTTCTTCACGATATGTGCATTCAACTCACAGAGTTGAAACTTTCTTTTCATAGAA
CACTTTTGAAACACACTTTTTGTAGAGTCTGCAACTGCATATCTGGAGCGCTTTGAGGCCTA
TGCTTGAAAAGGAAATATCTTCACATGAACACTAGACAGAAGCATTC

Monomer type t39

TCAGAAACTTCTTTGTGATGTGAGCATTCAACTCACAGAGTTGAACCTTCCTTTTGATAGAA
CAGTTTGGAAACACCCTTTTTAGAAAATCTGCAAGTGGATATTTCGAGCTTTTGGAGGCCTA
TGCTGGAAAAGAAAATATCTTCACATAAACCCTAGACAGAACCATTC

Monomer type t40

TCAGAAACTTCAGTTTGACGTGTGCATTTAACTCACAGTCTTCAACGTTCATTTTGATAGAA
CATTTTGGAAACACTCCTTTTGTGGAGTGTGCCAGGGCTTATTGTGAGCGCTTTGAGACCTA
TGTTAGAAAAGGAAATGTCTTCTTATAGAAACTAGACAGCAGCATTC

Monomer type t41

TCAGAAACTTCCTCGTGATGAGTGAATTCTACTCACAGAGCTGAACATTCCTTTGGATAGAA
CAGTTTTGAAACACTGTTTTTGTATAGTCTGCAAGTGGATAATTGGAGCGCTTTGAGGCCTG
TGCTGGAAAACGAAATATCTTCACATAAAAACCAGACAGAAGCATTC

Monomer type t42

TCAGAAACTTTTTCTCATGTGCATATTCAACTCACCGTGTTGAACCTGCCTTTTGATAGAGC
AGTTTTGAAACACTCTTTTTCTAGAATCTGCAAGTGTATATTGGAGTGCTAAGAGGCCAACA
ATGGGAAAGGAAATATCTTCCCATGAGAACTAGACGGAAGCATTC

Monomer type t43

TCAGAAACTTCCGTGTGCTTTATGCGTTTAACTCACATAGTGCAATCTTCCTTTAGATAGCG
CTTTTTTGAAACCCTCTTTTTGTAGAATTTGCTAGTGGATGTTTGGGGCTATTTGAGGCGTG
TGCTGCAAAAGGAAATATCTTCACATAAAAAGTAGACAGAAGCATTC

Monomer type t44

TCAGAAACTTCTTTGTCATGACTTCATTCAACTCACAGACTTGAAACTTCCTTTTGAAAGAG
GAGTTTTGAAACACTCTTTTTGTAGAATCTGCAAGTGGATATTTGGAGCGCTTTGAGGCCTA
TGCTGGGAAATGATATATCTTCACCTAAAAACTGGACAGAAGCATTC

Table S2. Canonical 7mer consensus sequence.

Monomer type t1
TCAGAAACTTCTTTGTGATGAGTGCATTCTACTCACAGCGTTGAAGCTTACTTTTGATAGAG
CAGTTTTGAAACACTCTTTTGGTAGAATCTGCAAGTGGATATTTGGAGCGCTTTGAGGCCTA
TGCTGGAAAAGGAAATGTCTTCACATAAAAACTAGACAGAAGCATTC

Monomer type t2
CAGGAAACTTCTGTATGACAAGTGCATTCAACTCACAGACTTGAACGTTCCTTTTGATAGAA
CTCTTTTGAAACACTCCTTTTGTAGAATTTGCAAGTGTTTATTTGGAGCGCTTTGAGGCCTA
AGCTGGAAAAGGAAATATCTTCACGTAAGAACTAGACAGAAGCATTC

Monomer type t3
TCAGAAACTTCTTTCTGATGAGTGCATTCAACACACAGACTTGAACCTTGCTTTTGAAAGAG
CAGCTTTGAAACACTCTTTTCGTAGAATCTGCAAGTGGATATTTGGAGCGCTTTGAGGCCTA
TGCTGGGAAATGAAATCTCTTCACCTAAAAACTAGACAGAAGCATTC

Monomer type t4
TGAGAAACTTCTGTGTGATGAGTGCATTCAACTCACAGACTTGAACGTTCCCTTTGATAGAA
CAGTTTTGAAACACTCCTTTTGTAGAATCTGCAAGTGTTTATTCGGAGCACCTTGAGGCCTA
TGTTGGAAAAGGAAATATCTTCACATAAAAACTAGAGAGAAGGATTC

Monomer type t5
TCAGAAACTTCTTTGTGATGAGTTCATTCAACTCACAGAGTTGAACCTACCAATTGATAGAG
AAGTTTGGAAACACTCTTTTTGTAGAATCTGCAAGTGGATATTTGGAGCCGTTTGAGGCCTA
TGCTGGAAAAGGAAATATCTTCACATAAAAACTGAACAGAAGCATTC

Monomer type t6
TCAGAAACTTCTTTGTGTTGAGTGCATTCAACTCACAGACTTGAACTTACCTATAGAAAGAG
CAGTTTTGAAACACTCTTTTGGTAAAATCTGCAAGTGGTTATTTGGAGCACTTCGAGGCCTG
TGCTGGGATATGGAATATCTTCACCTAAAAACTACAGAGAAGCATTC

Monomer type t7
TCAGAAAGTTCTTCACGATGCATTCATTCAACTCACAGAGTTGAACCTTCCTTTTGATAGAA
CAGTTTTGAAACACTCTTTTTGTAGAATCTGCAAGAGGATATTTGGAGCGTTTTGAGGCCTA
TGCTGGAAAAGGAAATATCTTCACATAAAAACTAGACAGAAGCATTC

Table S3. Canonical 18mer consensus sequence.

Monomer type t1
TGAGAAACTTCCCTGCGCTATTTGCATTCAACTCACACTCTTGAACCTTCTTTTTGAAAGAG
CAGATTTGAAACACTCTTTTTGTAGAAACTGCAAGTGCATATTTGGAGTGCTTTGAGGCCTA
TGATGGAAAAGGAAATATCCTCACAGAAAAATTAGACAAAAGCATTC
Monomer type t2
AAAGAAATATCTTCACATAATACCTAGACGGAAGCATTCTCAGAAACTTCTCTGTGATGAGT
CATTTATGAAACACTTTCTTTGAAGAATCTATAAGAGATTATTTGGAGCTCATTGAGGCCTA
TGCTGGAAAAAGGAATATCTTCATATAAAAACTAGACAGAAGCATTC
Monomer type t3
TCCGAAACTTCTTTGGGATGAATGCATTCAGCTCACAGAGTTGAACCTTGCCTTTGATAGAA
CAGTTTTGAAACACTCTTTTTTTAGAATCTGCAAGTGGATATTTGGAGCGCATTGGGACCTA
TGCTGGAA
Monomer type t4
TCAGAAACTTCCCTTTGCTTTATGCAGTCAACTCACAGACTTGAAACTTCCTTTTCAAAGAG
CAGATTTTAAATACTCTTTTTGTAGAAACTGCAAGTGGATATTTGGAGCGCTTTGAGGCCTA
TGCTGGAAAAGGAAATATCCTCACATAAAAACAAGACAAAAGCATTC
Monomer type t5
TCAGAGACTTCCTTGTGCCTTATACATTCAATTCACAGGCTTGAACCTTCTTTTTGGTAGAG
CAGTTTTGAAACACCCTTTTTGTAGACTCTGCAAGTGGATATTTACAGCGATTTGAGGCCTA
CAGTGAAAAAGGGGATATCTTCACATAAAAACTAGATAGAAGCATTC
Monomer type t6
TCAGAAATTTCTTTGTGATGAGTGCGTTCAACTCACAGGGTTGAACTTTCCTTTTGATAGAA
CAGTTTTGAAACACTCTGTTTGTAGAATTTGCAAGTGGATATTTGGAGCGATTTGAGGCCTA
TGCTGCAAAAAGGGAATATCTTAACATAAGAACTAGACAGAAGCATTC
Monomer type t7
TCAGAAACATTTTTGTGATGAGTGCATTCAACTCTGAGACTTGAACATTCCTTTTGAAAGAG
CAGTTTTGAAACACTCTTTTAATAGAATCTACAAGTGGGTATTTGGAGCAATTTGAGGCCTA
TGCTGGAAAAGGGAATATCTTCACATAAAAATTAGACAGAAGCATTC
Monomer type t8
TCAGACACTTCTCTGTGATGGGCGCATTCAGCTCACTGAGTTCAACCTTCCTTTAGGTAGAA
TAGTTTTGAATCACTCTCTTTGTAGAATCTACTATCGATTATTTGGAGCGCATTGAGGCCTA
CGATGGAAAAGGGAATATCTTCCCATAAAAACTAAGCAGAATCATTC
Monomer type t9
TCAGAAACATTTATGTGATGAGTGCTTTCAACTCAGAGACTTGAACATTCCTTTCGATAGAG
CAGTTTTGAAACACTCTTTTTGTAGAATCTGCAATTGGATATTTGGAGTGCTTTGAGACCTA
TGCTGGAAAAGGAAATATCTTCACATAAAAATTAGACAGAAGTATTC
Monomer type t10
TCAGACACTTCTCTGTGATGAGTGCATTCGACTCACAGAGTTCAACCTTCCTTTTGGTAGAA
CAGTTTTGAATCACTCTCTTTCTAGGACCTACAGTAGATTATTTGGAGCTCCTTTAGGCCTA
TGATGGGAAAGGGAATATCTTCCCATAAAAACTAAGCAGAATCATTC
Monomer type t11
TCTGAAACTTCTTTGTGATGAATGCATTCAGCTTACAGAGTTGAACTTTCCTTTTGATAGAA
CAGTTTTGAAACACTCTTTTTGTAGAATCTGCAAGTGGATATTTGGAGAGCTTTGAGACCTA
TGCTGGAAAAGGAAATATCTTCACATAAAAATTAGACAGAAGCATTC
Monomer type t12
TCAGAAACTTCAGTGTGCGTTATGCATTCAACTCACAGACTTGAACATTCCTTTTGAAAGAG
CAGATTTGAAAAACTCTTTTTGTAGAAATTGCAAGTGGATATTTGGAGTGGTTTGAGGCCTA
TGCTGGAAAAGGATATATCCTCACATAAAAACTAGACAAAAGCATTC
Monomer type t13

TCTGAAACTTCTTTGTGATGCGTGCATTCATCTAACAGACTAGAAACTTCCTTTTGATAGAG
CAGTTTTGAAACACCCTTTTTGTAGAATCTACAAGTGGATATTTGGAGCGATTTGAGGCCTG
TGGTGTAAAAGGGAATATCTTCACATAAAAACTAGAAGGAAGCATTC

Monomer type t14
TCAGAAACATCTCTATGATGAGCGCATTGTACTCACAGACTTGAACTTTCTTTTTGATAGAG
CAGTTTTGAAACACTCCTTTTGTAGAATGTGCAAGTGGATATTTGGTGTGCTTTGAGGCCTA
ATCTGGAAAAGGAAATATCTTCACATAAAAACTAGACAGAAGCATTC

Monomer type t15
TCACAGAGTTCTTAGTGATGAGTGCATCCAACTCACAGACTTAAACTTTCCTTTTGATATAG
CAGTTTTGACACACTCTTTTTGTAGAATCTGCAAGTGGATATTTGGAGCGCTATGAGGCCTA
CGCTGTAAAAGGAAATATCATCACATAAAAACTATACAGAAGCATTC

Monomer type t16
TCTGAAACTTCTTTGCGATGAGTGCATTCAACCCACAGAGTTGAAACCTCCTTTTTTTTAGA
ACTGTTTTGTAACACTCTCTTTGTAGAATCTGAAAGTGGATACTTGGTGCGCTTTGAGGCCT
ATGCTGGAAATGGAAATATGTTCTCATAAAAACTAAGCAGAAGCATAC

Monomer type t17
CCAAAAACTTCCCCGTGCTTCATGCATTCAACTCACAGACTTGAACCTTCCTTTTGATAGAG
CAGTTTTGAAACACTCTTTTTGTAGAATCTGCAATTGGATATTTGGAGCGATTTGAGGCCTA
TGATGAAAAAGGGAATGTCTTCACCTAAAAACTAGACAGAAGCATTC

Monomer type t18
TCAGAAATTTCTTTGTGATGTGTGCATTTAATTCACCGAGTTGAACTTTCCTTTTGATAGAA
CAGTTTTGAAACAGTCTTTTTGTAGAATCTGCAAGTGGATATTTGGAGCATTTTGAGGTCTA
TGCTGGAAAAGGAAATATCTTCACATAAAAACTAGACAGAAGCATTC

Monomer type t19
TCAGAAATTTCTGTGTGATGAGTGCATTCAACTCACAGGGTTGAACTTTCCTTTTGCTAGAA
CAGTTTTGAAACATTCTTTTTGTAGAATCTGCAAGTGTGTATTTGGAGCGATTTGAGGCCTA
TGCTGCAAAAAGAGAAATCTTCACATAAAAACTAGACAGAAGCATTC

Monomer type t20
TCAGAAATATTTTTGTGATGAGTGCATTCAACTCACAGCCTTGAACCTTCCTTTTGATAGAG
TAGTTTTGAAACACTCGTTTTGTAGAATCTGCAAGTGGATATTTGGAGTGCTTTGAGGTCTA
TGCTGGAAAAGGAAATATCTTCACATCAAAACTAGACAGAAACATTC

Monomer type t21
ACAGAAACTTCTCTGTGATGAGTGCATTCACCTCACAGAGTTCAACCTTCCTTTTGGTAGAA
CAGTTATGAAACACTCTCTTTGTAGAGTCTACAAGAGATTATTTGGAGCTCATTGAGGCCTA
TGCTGGAAAAGAAAGTATCTTCCCGGAAAAACTAAGCAGAATCATTC

Monomer type t22
TCCGAAACTTCTTTGTGATGAATGCATTCACCTCACAGAGTTGAACCTTCCCTTTGATGGAA
CAGTTTTGAAACACTCTTTTTGTAGAATCTGCAAGTGGCTATTTGGAGCGCTTTGAGGCGTA
TGCTGGAAAAGGAAATATCTTCACATAATAAATAGACGGAAGCATTC

Monomer type t23
TCAGAAACTTCCCTGTGCCGTATGCATTCAACTCACGGACTTAAAACTTCCTTTTGAAAGAG
CAGATTTGAAACACTTTTTTTGTAGAAACTGCAAGTGGATATTTGGAGCGCTTTGAGGCCTA
TGCAGGAAAAGGAAATATCCCCACATAAAAAGTAGACAAAAGCATTC

Monomer type t24
TCCGTATTTTCTTTGTGATGTGTGCATTAAACTAACAGAGTAGAACCTTCCTTTTGATAGAG
CAGTTTTGAAACCCTCTTTTTGTAGAATCTGCAAGTGGATATTTGGAGCGATTTGAGGCCTA
TGGTGAAAAAGGGGATATCTTCGCATAAAAACTAGATAGAAGCATTC

Monomer type t25
TCAGAAACTTCTTTGTGATGAGTGCATTGTACTCACAGACTTTAAATTTCCTTTTGATAGAG
CAGTTTTGAAACAGTCTTTTTGTAAAATATGAAAGTGTATATTTGGTGCGATTTGAGGGCTA
AGATGGAAAAGGAAATATCTTCACATAAAAACTGGACAGAAGTATTC

Monomer type t26
TCAGAAACTTCTCTGTGTTTTATGATTTCAACTCACAGAGTTGAACCTTCCTTTTGATAGCG
CAGTTTTGAAACACTCTTTTGTAGAATCTGCAAGTGGATATTTGGAATGCTTTGAGGCCTAT
GCTCGAAAAGGAAATTTCTTCATATAAATACTAGACAGAAGCATGC
Monomer type t27
TCAGAAACTTCTTTGTGATGTGTGCACTCAACTCACAGATTTGAACCCTGCTTTTCAAAGAG
CAGTTATGAAACACCCTTTTTGTAGGATCTGCAGGTGGACATTTTTGGCGCTTTGAGGCCAA
TGCTGGAAAAGGAAATATGTATACATAAAAACTAAACAGAAGCATTC
Monomer type t28
TCAGAAACTTCCCTGTGCTTTATGAATTCAACTCACAGACTTGAACCTTCCTTTTGATAGAA
CAGTTTGGAAAAACACTTTTTGTAGAATCTGGAAGTGGATATTTAGAGCGCTTTGAGGCCTA
TGCAGGAAAAGGAAATACCATCACATAAAAACTAGACAGAAGTATTC
Monomer type t29
TCAAAAACATCTTTGTGATGTGTGCCTTCAACTCACAGAATTGAAACTTCCTTTTGATAAAG
CAGTTTTGAAACACCATATTGTAGAATCTGCAAGTGGATAGTTTTAGCACTTTGAGGCCTAG
CCTGGAAAAGGGTATATCTTCACATAAAAACTGGACAGAAGCATTC
Monomer type t30
TCAGAAACTTCTTTGTGATGAGTGCATTCAACTCACAGTGTTGAAAGTTCCTTTTGATAGAA
CAATTGTGACACAATCTTCTTATAGAATCTGCAAGTGGATATTTGGATTGCTTGAGGCCTAT
GCTGGAGTAAGAAATAATTTCACATAAAAACTATATTGAAGCATTC
Monomer type t31
TCAGAAACTTCTGTGGGATCAGAACGTTCAACCAACAGAGTTGAACCTTCCTTTTGATAGAC
TAGTTTTGAAACACACTTTTTGTAGAATCTGCAAGTGGATGTTTGGAGGGCTTTGAGGCCTA
TGCTGGAAAGAAAATATCTTCACCTAAAAACTAGACAGAAGCATTC
Monomer type t32
TCAGAGACTCCCCTGTGCTTTATGCATTCAAATCACAGGCTTGAACCTTCCTTTTGATAGAG
CAGTTTTGAACCACTCTTTTTGTAGAATCTGCAAGTGGATATTTGGAGTGATTTGAGGCCTA
TGATGAAAAAGGAAATATCTTCACCTAAAAACTAGACAGAAACATTC
Monomer type t33
TCAGAAATTTCTTGGTGATGAGTGCATTCAACTCACAGGTTTGAACTTGCCTTTTGATAGAA
CAGCTTTGAAACACTCTGTTTGTAGAATTTGCAAGTGGATATTTGGAGAGATTTGTGGCCTG
TGCTGCATAAAGGGAATATCTTAACTTTAGAACAAGACAGAAGCATTC
Monomer type t34
TCAGAAATTACTTCGGGATGAGTGCATTCAACCCAAAGAGTTGAACCTTCCTTTTGATAGAA
CAGTTTTGAAACACTTTTTTTGGAGAATCTGCAAGTGGATATTTGGAGCAATTTGAGGCCTA
TGCTAGAAAAGGAAATATCTTCACATAAAAACTAGACGGAAGCATTC
Monomer type t35
TGCGATACCTTTTTGTGATGAGTGCATTCAACTCACAGACTTGAACATTCCTTTCGATAGAG
CAGTTTTGAAACACTCTTTTTGTAGATTCTGCAAGTGGGTATTTTGAGCGCTTTGAGGTCAA
AGATGGAAAAGGAAATATCTTCATATAAAAACTAGACAGAAGCATTC
Monomer type t36
TCAGACACTTCTCTGTGATGAGTGCATTCAACTCACAGAGTTCAACCTTCCTTTTTGTAGAA
CAGTTATGAAACACTCTCTTTGTAGAATCTACAAGGGATCATTTGGAGCACATTAAGGCCTA
TGCTGGAAAAGGAAGTATCTTCCCATAAAAAAAAAAGCAGAATCATTC
Monomer type t37
TGAGATACTTTTTTGTGATCAGTGCATTCGACTCACAGACTTGCACCTTCCTTTTGATAGAG
CAGTTTTGAAACACTCTTTTTGAAGAATCTTCAAGTGGATATTTGGAGCGCTTTGAGGCCTA
TGCTGGAAAAGGAAATATCTTCACATAAAAACTAGACAGAAGCATTG

Table S4. Canonical 14mer consensus sequence.

Monomer type t1
TGTGAAACTGGTTTGTGTAGCGTGAATCCAACTCACAGAGTTGAAAAACTTTTTCTGAAAGA
GCAGTTTTGAAATATTCTTTGTAGAATGTGCAATTGTTCATTAGGAGCACTTTGAGGCCTAT
GTTGGAAAAGGAAGTATCTTCACAAAAAATCCAGACAGAAGCATTC
Monomer type t2
TCAGAAACAATTTTTTGATGTGTGCATTCAACTCACAGAGTTGAACCTTCTTTTTCTTAGAG
CAGTTTTGAAACAGTGTTTTTCCAGAATCTGCAAGTGGATATTTGGAGCACTTACAGGCCAT
CGTTGGAAATGGTAACATCTTCAAATAAAAACTAGATGGATGCATTC
Monomer type t3
TCAGAAACTGCTTCGTGTTGTATGCCTTCAACTCACAATGTTGAATCTTTCTTTTGATAGAG
CAGTTTTGAAACACTCTTTTTTTGTAGAATCTGCAAGTGTTCATGGGGTGTGCTTTGAGGTC
TATCGTGGAAAAGGAAATATCTTCATATAAAAACTAGACAGAAGCATTC
Monomer type t4
TCAGAAACACCTTTATGGTGTGTGCATTGAACTCAGAGAGATAATCCTTCCTTTTGATAGAG
CAGTTTTGAAACAGTGTTTCTGCAGAATATGCAAGTGGACATTTGGATCTCTTGAGGCCTTC
GTTGGAAACCAGAATATCTCACACAAAAAAACTAGACAGAAGTGTTC
Monomer type t5
TCAGAAACTGCTTTGTGATGTGTTTCTTCAACCCACAGAATTGAACATTTCCCTTGATAGAA
CAGTTTTGAAACACCCTTTTTGTAGAATCTGCAAGTTTTCTTTTGGAGGGCTTTGTGGCCTA
TGGTGGAAAAGGAAATATCTTCATATAAAAACCAGACAGAAGGTTTC
Monomer type t6
TCTGAAACTTCTTTACGATGTGTGCATTCAACTCACAGACTTGAAACTTCCTTTTCGTGGAG
CAGTTTTCAAAGCGTCATTTTGCAGAATCTGCAAGTGGATATTTGTAGCTCTTTCAGGCTTT
CGTTGGAAACGGGAATATCTTCACATAAATACTGGACAGCAGCATTC
Monomer type t7
TCTGAAACTACTTTCTGTTGTATGCGTTCGTCACCCAGAGTTGAATCTGTATTTTGATAGAA
CAGTTTTGGAAAAGTCTTTTTGTAGAATCTGCAAGTGTTCATTTGGTATGCTTTGAGGCCAA
TGGTGGAAACGGAAATACTTTCACATAAAATCTAGACAGAAGCATTC
Monomer type t8
TCTGAAATTTCCTTATGATGTGTGCATTCAACAAACAGAGTTCAACCTTCCTTTGGGTAGAG
CAGTTTTGAAATAGTATTTTTGCAGAATCTGCATAAGGATATATATGGAGCTCTTTGAAGCG
TTCGTTGGAAACGGGACTATCTTCACATAAAAACCAGACAGAATTGTTC
Monomer type t9
TCAGAAACTGCTTTGTGTTGCGTGCATGCAACACACAGAGTTGAACCTTTCTTTTGATAGAG
CACTTTTGAAACACTCTTTTTGTAGAATTTGCAAATGTTCATTAGGAGCGCTTTGAAGCCTA
TGGTGGAAAAGGAAACAACTTCACAGAAAAACTAGACAGAAGCATTT
Monomer type t10
TCTGAAACTTCTTTAAGATGTGTGTATTCAACTCAGAGACTTGAACCTTCCTTTCGATGGAG
CAGTCTTGAAAGAGACTTTTCGCAGAATCTGCCAGTGGATATTTGGAGCTCTTTGAGACCTT
CGTTGGAAACGTGAATATCTTCACATAAAAACTAGACAGAAGTGTTC
Monomer type t11
TCTGAACCTGCTTTGTGTTGTACACATTCAACTAACAGAGTTGAATCTTTCTTTTAATACAG
CAGTTTTGAAACACTTTTTTTTATAGAATCTGCAAGTTTTCATTCGTTGTGCTTTGAGGCCA
ATGGTGGAAAAGGAAATATCTTCACATAAAATCTAGACAGAAGCATTT
Monomer type t12
TAAGAAACTCCTTTATGACGTGTGCATTTAACTAACAGAGTTGAATCTTCCATTGTTTGAGC
AGTTCTGAAACAGTGTTTTTGCAGAATATGCATGTGGATATTTGGAGCTTTTTGAGGCGTTC
GTTGGAAATGGTACAATCTTCACATAAAACTAGACAGAAGTGTTC
Monomer type t13

TCAGAAACTTCTTTGTGTTGTGTGCACTCAACACACAGAGTTGAACCTTTCTTTTGATAGAG
CACTTTTCAAACACTCTTTTGGTAGAATTTGCAAGTGTTCATTTGGAGGGAATTGAAGCCCA
CGGTGGAAAAGGAAACAATGTCACATAAAAACATGACGGAAGCATTC

Monomer type t14

TGTGAAAGTTATTTATGATGTGTGCTTTCAACTCACAGAGTTCAACCTTCCTTTTGAAAGAG
CAGTTTTGAAACATTCTTTTTATGTTATCTGCAAGTGGATATTTGGAGCTCTTTGAGGCCTT
TGTTGGAAACGTGAATATCTTCACATAAATACTAGACAGAGGTATTC
