# Supplementary material for: Cascading 58mer Alpha Satellite superHOR in Complete Orangutan Y Chromosome
Source: Int J Mol Sci. 2025 Aug 22;26(17):8122. doi: 10.3390/ijms26178122 (PMC12428378; doi:10.3390/ijms26178122)

Fig. S1. Cascading 58mer alpha satellite HOR alignment. Start position 18,488,201 bp and end position 21,586,501 bp in NHGRI_mPonAbe1-v2.0 chromosome Y. The number on the left side indicate the initial position of the first monomer in each row of HOR copy.

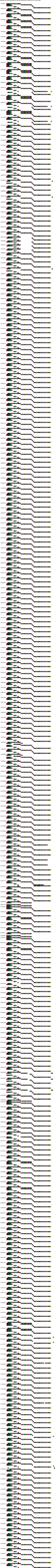

Supplement: Supplementary file 1 [file FigS1.pdf › FigS1.pdf]
